# The Kaposi’s Sarcoma-Associated Herpesvirus (KSHV) gH/gL Complex Is the Predominant Neutralizing Antigenic Determinant in KSHV-Infected Individuals

**DOI:** 10.3390/v12030256

**Published:** 2020-02-26

**Authors:** Yasaman Mortazavi, Salum J. Lidenge, Tara Tran, John T. West, Charles Wood, For Yue Tso

**Affiliations:** 1 Nebraska Center for Virology and School of Biological Sciences, University of Nebraska-Lincoln, Lincoln, NE 68588, USA; yass.mortazavi@yahoo.com (Y.M.); sjlidenge@yahoo.co.uk (S.J.L.); tara921m@gmail.com (T.T.); jwest2@unl.edu (J.T.W.); 2 Ocean Road Cancer Institute, Dar es Salaam 3592, Tanzania; 3 Muhimbili University of Health and Allied Sciences, Dar es Salaam 65001, Tanzania

**Keywords:** KSHV, KS, glycoproteins, gH, gL, neutralizing antibodies

## Abstract

Kaposi’s sarcoma-associated herpesvirus (KSHV) is the etiological agent of Kaposi’s sarcoma (KS), one of the most prevalent cancers of people living with HIV/AIDS in sub-Saharan Africa. The seroprevalence for KSHV is high in the region, and no prophylactic vaccine against the virus is available. In this study, we characterized the antigenic targets of KSHV-specific neutralizing antibodies (nAbs) in asymptomatic KSHV-infected individuals and KS patients with high nAbs titers. We quantified the extent to which various KSHV envelope glycoproteins (gB, ORF28, ORF68, gH, gL, gM, gN and gpK8.1) adsorbed/removed KSHV-specific nAbs from the plasma of infected individuals. Our study revealed that plasma from a majority of KSHV neutralizers recognizes multiple viral glycoproteins. Moreover, the breadth of nAbs responses against these viral glycoproteins varies among endemic KS, epidemic KS and asymptomatic KSHV-infected individuals. Importantly, among the KSHV glycoproteins, the gH/gL complex, but neither gH nor gL alone, showed the highest adsorption of KSHV-specific nAbs. This activity was detected in 80% of the KSHV-infected individuals regardless of their KS status. The findings suggest that the gH/gL complex is the predominant antigenic determinant of KSHV-specific nAbs. Therefore, gH/gL is a potential target for development of KSHV prophylactic vaccines.

## 1. Introduction

Kaposi’s sarcoma-associated herpesvirus (KSHV), also known as human herpesvirus-8 (HHV-8), is the causative agent of all forms of Kaposi sarcoma (KS) and two additional lymphoproliferative diseases, primary effusion lymphoma (PEL) and multicentric Castleman’s disease (MCD) [1,2]. The distribution of KSHV varies globally. In some areas such as sub-Saharan Africa (SSA), KSHV seroprevalence can be as high as 80%, whereas in the US and Europe, the prevalence is 3%–20% [3,4]. The prevalence of KS increases significantly with HIV-1 infection, making it one of the leading cancers among people living with HIV/AIDS in SSA [5,6]. Globally, it is estimated that nearly 44,000 new cases of KS emerge annually, with the highest incidence occurring in Africa, where KSHV is endemic [7]. The two main clinical manifestations of KS are Endemic KS (EnKS) and Epidemic KS (EpKS). EnKS is found among HIV-1 negative individuals, whereas EpKS is associated with HIV-1 co-infection [8,9]. Additionally, in specific ethnic groups of elderly men of Mediterranean or Eastern European descent, there exists Classical KS (CKS), and Iatrogenic KS (IKS) is associated with drug-induced immune suppression [10]. The recurrence rate for KS is high even with chemo- or radiotherapy [11]. Therefore, the most cost-effective strategy to reduce KS incidence is to prevent KSHV infection through vaccination, and understanding the antigenic determinant of KSHV-specific nAbs will contribute to informative vaccine research for KSHV.

Although KSHV-specific nAbs have been reported in KS patients and KSHV-infected individuals, their role in KSHV pathogenesis is not known [12,13]. More importantly, it is unclear whether having KSHV-specific nAbs prior to exposure to KSHV could be beneficial in preventing its infection and therefore could serve as an adaptive correlate of protection against KS development. Based on lessons learned from vaccination against other viruses, such as influenza, nAbs elicited through vaccination primarily target viral membrane surface proteins [14,15]. It has also been shown that immunization with KSHV envelope glycoproteins gB, gpK8.1 and gH/gL can elicit nAbs responses in animal models [16,17]. However, whether these glycoproteins are the targets for nAbs elicited through infection, as in KSHV-infected individuals, and whether these glycoproteins can induce nAbs in immunized humans, remain to be determined.

Herpesviruses initiate infection via binding to various receptors on the surface of target cells. This process is primarily mediated by multiple viral glycoproteins embedded in the viral envelope [18]. These glycoproteins play important roles in virus attachment to target cells and fusion of the viral envelope with either cytoplasmic or endosomal membranes, as well as the virion morphogenesis and egress [19]. KSHV incorporates eight glycoproteins into its virion envelope: ORF8 (gB), ORF28, ORF68, ORF22 (gH), ORF47 (gL), ORF39 (gM), ORF53 (gN) and gpK8.1 [20]. Studies have shown that distinct envelope glycoproteins are involved in KSHV infection of different cell types, with gpK8.1, gB, and gH/gL as the major players [21,22,23]. The current model for KSHV infection of most cell types suggests that virion binding is mainly initiated via gB or gpK8.1 interactions with the host cell heparin sulfate (HS) or integrin proteins, which then trigger gH/gL engagement with the ephrin receptor tyrosine kinases leading to fusion [24,25,26,27,28,29,30]. Given the critical roles of KSHV glycoproteins in viral entry, we hypothesized that KSHV glycoproteins could play a pivotal role in the elicitation of KSHV-specific nAbs in infected individuals. To test this hypothesis, we studied the ability of various KSHV glycoproteins in the adsorption/removal of KSHV-specific nAbs from the plasma of KSHV-infected individuals who have high nAbs titers. Glycoprotein targeted by the nAbs could be identified by a reduction in the nAbs response after depletion with a specific glycoprotein.

With this approach, we have characterized the variable specificity of nAb responses and demonstrated that multiple KSHV glycoproteins are the targets of KSHV-specific nAbs in KSHV-infected individuals regardless of their KS status. The results reveal that the gH/gL complex depleted the highest amount of KSHV-specific nAbs in the majority of KSHV-infected individuals, suggesting that gH/gL is the most prominent target of KSHV-specific nAbs, and therefore the most plausible antigenic target for prophylactic vaccine development against KSHV.

## 2. Materials and Methods

### 2.1. Ethics Statement

Written informed consent was obtained from all study participants from Tanzania and Zambia for sample collection and testing. The study was conducted in accordance with the Declaration of Helsinki and approved by the review boards of Tanzania National Institute for Medical Research, Ocean Road Cancer Institute, University of Zambia Biomedical Research Ethics Committee and the University of Nebraska-Lincoln (Tanzania CRITIC: IRB Number: 20141014709FB, 10/25/2019 and Zambia ZAMDAPP: IRB Number: 20170817442FB, 08/09/2019).

### 2.2. Cell Cultures

Human embryonic kidney T (293T) and BC-3 cells were obtained from ATCC (CRL-3217 and CRL-2277, respectively). 293T cells were maintained in Dulbecco’s modified Eagle medium (DMEM) with 10% fetal bovine serum (FBS) and 1% penicillin–streptomycin (P/S). BC-3 cells were maintained in RPMI with 20% FBS and 1% P/S. Vero.219 cells (a gift from Jeffrey Vieira [31]) were maintained in DMEM with 10% FBS and 1% P/S and 6 µg/mL of puromycin. All cell cultures were maintained at 37 °C in a 5% CO_2_ incubator.

### 2.3. KSHV Serological Assays

The KSHV serostatus and total anti-KSHV antibody titers were determined by immunofluorescence assay (IFA) on activated BC-3 cells as previously described [32]. To determine the total KSHV-specific nAbs titer, neutralization assays were carried out in triplicate as previously described [13]. Briefly, heat-inactivated plasma (56 °C for 1 h) from study participants was incubated with rKSHV.219 virus at 1:50 dilution at 37 °C for 1 h. 293T cells seeded in 96-well plates (2.5 × 10^4^ cells/well) were infected with the virus–plasma mixture, centrifuged (400× *g* for 20 min) and then incubated for 72 h at 37 °C. Neutralization activity of the plasma, relative to a KSHV negative plasma, was then quantified by flow cytometry at 72 h postinfection as follows:

*%neutralization* = 100 − [(s/c) × 100] (1)

S = % GFP positive cells in wells with KSHV positive plasma.

C = % GFP positive cells in wells with KSHV negative plasma.

Plasma samples that were positive for KSHV nAbs at the 1:50 dilution were further titrated by 2-fold dilutions from 1:50 to 1:3200 to define the IC_50_ (50% inhibitory concentration).

### 2.4. HIV-1 Serology and Plasma Viral Load Quantification by Real-Time PCR

The HIV-1 diagnosis was made according to Alere Determine HIV-1/2 Ag/Ab Combo test in Zambia and Tanzania HIV Rapid Test Algorithm. The HIV-1 serology results were verified using HIV-1–2.0 First Response kit (Premier Medical Corporation Ltd., Mumbai, India). To quantify HIV-1 plasma viral load, the viral RNA was extracted from plasma following the QIAamp viral RNA extraction protocol (Qiagen, Hilden, Germany) and measured using the RNA Ultra-Sense One-Step quantitative real-time PCR (qPCR) system (Applied Biosystems, Foster City, CA, USA) as previously published [33].

### 2.5. KSHV Virion in Plasma

Plasma of study participants (400 µL) was centrifuged at 8000× *g* at room temperature for 10 min to remove residual cells. Then, 15 µL of DNase-I (Qiagen, Hilden, Germany) was added to each sample and incubated for 2 h at room temperature to digest cell-free genomic DNA. The samples were then incubated at 65 °C for 20 min to inactivate DNase-I, and viral DNA was extracted by the QIAamp DNA mini kit according to the manufacturer’s protocol (Qiagen, Hilden, Germany). The presence of KSHV virion in plasma was then determined by nested PCR of the extracted viral DNA using primers for the open reading frame 26 (ORF26) amplicon (forward 5′-AGCCGAAAGATTCCACCAT-3′ and reverse 5′-TCCGTGTTGTCTACGTCCAG-3′ in the first round and forward 5′-CGAATCCAACGGATTTGACCTC-3′ and reverse 5′-CCCATAAATGACACATTGGTGGTA-3′ in the second round reaction) under conditions previously described [34]. Contamination of the extracted viral DNA by cell-free genomic DNA was ruled out through a negative PCR result for the human β-actin gene.

### 2.6. KSHV Envelope Glycoprotein Constructs

To construct the KSHV gB, gpK8.1, gH/gL, gM, gN, ORF28 and ORF68 3XFLAG-tagged plasmids, the full-length open reading frame sequence of each individual glycoproteins (NCBI reference sequence #NC_009333.1) was PCR-amplified from BC-3 genomic DNA and cloned into the pcDNA3.1 mammalian expression vector (Invitrogen, Carlsbad, CA, USA) with a 3xFLAG fused to the carboxyl-terminus of each glycoprotein. The resulted plasmids were confirmed with restriction enzyme digestions and sequencing.

### 2.7. Immunoblot

1 × 10^6^/well of 293T cells were seeded into 6-well plates. At 24 h postseeding, 2 µg of each KSHV glycoprotein expressing plasmids were transfected into the 293T cells using FuGENE 6 transfection reagent (Promega, Madison, WI, USA). After 72 h, the transfected cells were lysed in RIPA lysis buffer with proteinase inhibitor cocktail (Thermo Scientific, Waltham, MA, USA). The extracted protein was then measured by Pierce BCA protein assay (Thermo Scientific, Waltham, MA, USA) and an equal amount of protein from each glycoprotein transfected cell culture was loaded and resolved in a 4%–15% gradient SDS-PAGE (Bio-Rad, Hercules, CA, USA). The proteins were then transferred onto a nitrocellulose membrane and blocked with 5% skim milk in 1X PBS with 0.5% Tween 20 for 2 h at room temperature. The membranes were then incubated with either mouse monoclonal anti-FLAG M2 antibody (Sigma-Aldrich, St. Louis, MO, USA) at 1:100 or mouse monoclonal anti-GAPDH (6C5) IgG (Santa Cruz Biotechnology, Dallas, TX, USA) at 1:2000 overnight at 4 °C. The next day, after washing, the membranes were incubated with donkey anti-mouse 800CW or 680CW antibodies (Li-Cor Biosciences, Lincoln, NE, USA) at 1:10000 for 2 h at room temperature and then washed repeatedly. The membranes were then visualized with Odyssey infrared imager (Li-Cor Biosciences, Lincoln, NE, USA).

### 2.8. Immunohistochemistry (IHC)

1 × 10^4^/chamber of 293T cells were seeded into 4-well chamber slides. At 24 h postseeding, equal copy number of KSHV glycoprotein-expressing plasmids were transfected into 293T cells using FuGENE 6 transfection reagent (Promega, Madison, WI, USA). After 72 h, the slides were washed and fixed in 4% paraformaldehyde for 30 min at room temperature. The fixed slides were then incubated in 0.3% H_2_O_2_ methanol solution for 30 min at room temperature, followed by washes and antigen retrieved in sodium citrate solution for 15 min at 98 °C. After cooling to room temperature, the slides were washed and blocked with 10% normal goat serum for 30 min at room temperature and incubated with mouse monoclonal anti-FLAG M2 antibody (Sigma-Aldrich, St. Louis, MO, USA) at 1:300 in blocking solution at 4 °C overnight. The next day, the slides were washed and incubated with Dako EnVision+ System-HRP anti-mouse polymer for 30 min at room temperature, and color was developed with DAB solution (Dako, Santa Clara, CA, USA). Counterstaining of the nucleus was performed using hematoxylin. The final stained slides were then air-dried and rinsed twice in xylene solution for 5 min each, and coverslip was added with Cytoseal 60 (Thermo Scientific, Waltham, MA, USA). The slides were examined with a Nikon Eclipse 50i microscope under 20X magnification. Three times 3 fields of pictures (i.e., 9 pictures) were taken at 20X magnification and digitally stitched using NIS-Elements imaging software (Nikon, Tokyo, Japan).

### 2.9. Immunofluorescence Assay for Protein Expression

5 × 10^4^/chamber of 293T cells were seeded into 8-well chamber slides. At 24 h postseeding, equal copy numbers of KSHV glycoprotein expressing plasmids were transfected into 293T cells using FuGENE 6 transfection reagent (Promega, Madison, WI, USA). After 72 h, the slides were washed and fixed in 4% paraformaldehyde for 30 min at room temperature. The fixed slides were incubated with a heat-inactivated (56 °C for 1 h) pooled plasma from all the study subjects at 1:40 at 4 °C overnight. The next day, the slides were washed and incubated with mouse monoclonal anti-human antibody (ATCC, CRL-1786) at 1:4 at 37 °C for 1 h. Then the slides were washed and incubated with CY2 AffiniPure donkey anti-mouse IgG (H+L) antibody (Jackson ImmunoResearch, West Grove, PA, USA) at 1:200 at 37 °C for 1 h. Next, the slides were washed and stained with 0.001% Evans blue for 5 min at room temperature. After staining, the slides were washed and coverslip was mounted with mounting media. The slides were examined with a Nikon Eclipse 50i microscope under 20X magnification.

### 2.10. rKSHV.219 Production

Vero.219 cells, a cell line latently infected with a recombinant KSHV encoding the green fluorescent protein, were used to generate rKSHV.219 viruses. Briefly, a total of 18 × 10^6^ Vero.219 cells per flask were seeded into T-225 flasks. At 24 h postseeding, the culture medium was removed, and 3300 viral particles of adenovirus type 5 encoding KSHV RTA (Ad5-RTA) (gift from Dr. Jean Gustin) were incubated with the cells in the presence of 5M valproic acid without (w/o) puromycin. The next day, the cells were washed and fresh medium w/o valproic acid and puromycin was added. After 48 h, the supernatant was collected and was cleared by a 10 min spin at 2000 rpm. Then, the supernatant was collected and concentrated through a 20% sucrose cushion by spinning at 28000 rpm for 2 h. The virus pellet was resuspended in medium and titered on 293T using flow cytometry for measuring GFP positive cells to determine the amounts of virus needed to achieve 40%–50% GFP positive cells in the neutralization assays (Supplementary Figure S1A).

### 2.11. KSHV Glycoproteins Transfected 293T Cells Adsorption of nAbs from Plasma

Thirteen µL of the heat-inactivated plasma (56 °C for 1 h) was incubated with 1 × 10^6^ 293T cells (transfected with equal copy number of KSHV envelope glycoprotein plasmids either individually or in combination) in 578 µL volumes of medium (DMEM containing 10% FBS, 1% P/S and 1% protease inhibitor cocktail) at 4°C overnight. The number of cells and temperature used for adsorption were optimized based on the gH/gL complex (Supplementary Figure S1B,C). After incubation with the KSHV glycoproteins expressing 293T cells, the plasma-cell mixture was spun down at 6000× *g* for 5 min to remove the cell pellets, and the supernatant was collected. Then, neutralization assays were performed in triplicate, the supernatant was incubated with 59 µL of rKSHV.219 at 37 °C for 1 h. 293T cells in 96-well plates (2.5 × 10_4_ cells/well) were then infected with virus–plasma mixture, centrifuged (400× *g* for 20 min at room temperature) and incubated for 72 h. Neutralization was quantified by flow cytometry at 72 h postinfection. The percentage of neutralization was defined as follows:

%*neutralizatin* = 100 − [(s/c) × 100] (2)

S = % GFP positive cells in wells with KSHV glycoproteins absorbed sample plasma.

C = % GFP positive cells in wells with pooled plasma from HIV-1 and KSHV double-negative donors (*n* = 10).

The percentage of relative nAbs adsorption was defined as follows:

Relative % *nAbs adsorption =* [(B − N)/B] × 100 (3)

N = % neutralization of KSHV glycoproteins absorbed sample plasma.

B = % neutralization of sample plasma absorbed with empty-vector-transfected 293T cells.

### 2.12. Statistical Analysis

Statistical analyses were carried out using GraphPad Prism 5 (GraphPad Software Inc, San Diego, CA, USA). The comparison of nAbs responses to gH/gL complex with other KSHV glycoproteins among all the participants was performed using the Mann–Whitney test. The comparison of nAbs responses to each KSHV glycoprotein in the EnKS, EpKS and non-KS groups was performed using the Mann–Whitney test. Correlation between the extent of nAbs absorbed by gH/gL and total anti-KSHV and nAbs responses was performed using the nonparametric Spearman correlation analysis.

## 3. Results

### 3.1. Study Cohort

The study cohort was comprised of 25 individuals from Zambia and Tanzania: 21 KS patients and 4 asymptomatic KSHV seropositive participants. Among the KS patients, there were 15 EpKS and 6 EnKS individuals, while the non-KS participants consisted of one HIV-1^-^ and three HIV-1^+^ individuals (Table 1). The age of the study subjects ranged from 24 to 68 years old, with a median of 27 years for EnKS, 41 years for EpKS and 39 years for non-KS participants. All the EnKS cases were male, while 73.3% of the EpKS cases were male, and all the non-KS cases were female (Table 1). The duration of symptomatic KS ranged from 2 months to 108 months, with a median of 12 months for EnKS and 24 months for EpKS. The majority of the EpKS patients were on anti-retroviral treatment and had HIV-1 plasma viral load below detection limits. The 1/IC_50_, which indicates the dilution of plasma that accomplish 50% inhibition of KSHV infection, was performed using neutralization assays to determine the KSHV-specific nAbs titers in the plasma. The KSHV serostatus and total anti-KSHV antibody titers for all participants were determined by immunofluorescence assay (IFA). Both the total anti-KSHV antibody and KSHV-specific nAbs titers were variable among KSHV-infected individuals. For the available samples, the presence of KSHV virions in plasma was analyzed by PCR for the KSHV ORF26 gene. All participants in this cohort are high neutralizers as defined by our flow cytometry-based neutralization assay (supplementary Figure S2).

### 3.2. KSHV Envelope Glycoprotein Expression in 293T Cells

**Figure 1:**
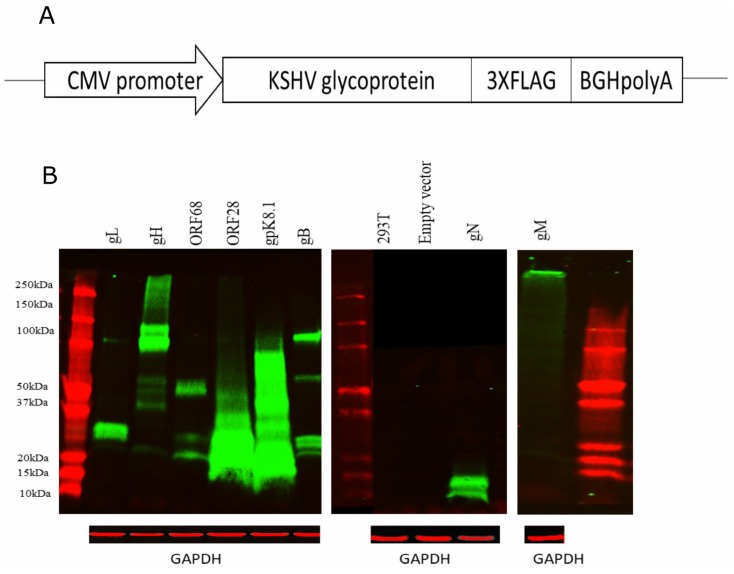
Kaposi’s sarcoma-associated herpesvirus (KSHV) envelope glycoprotein constructs and their expression in 293T cells by immunoblot. (**A**) Schematic representation of pcDNA3.1 mammalian expression vector encoding KSHV glycoproteins fused with 3xFLAG at the C-terminus of each glycoprotein (not drawn to scale). (**B**) KSHV glycoproteins expression was demonstrated in the whole-cell lyses of transfected 293T cells by immunoblot against the 3xFLAG tag.

Plasmids encoding KSHV envelope glycoproteins gB, ORF28, ORF68, gH, gL, gM, gN and gpK8.1 were generated by cloning the full-length sequence of each gene into the pcDNA3.1 mammalian expression vector (Figure 1A). To facilitate the detection of these glycoproteins, the sequence for the 3xFLAG epitope was fused to the carboxyl-terminus of each glycoprotein construct, the expression of which was evaluated after transfection of 293T cells. At 72 h post-transfection, high expression levels of all KSHV glycoproteins were detected in the whole-cell lysates of their respective transfected cells using immunoblotting against the 3xFLAG tag, demonstrating that all the KSHV glycoprotein-expressing constructs are functional (Figure 1B). However, since the input plasmids transfected into the cells was based on total DNA amount, rather than equal copy number, there is some variation in expression among different glycoproteins. For most of the KSHV glycoproteins, their corresponding bands, as reported by other groups, were detected by immunoblot in the total cell lysates [17,20,22,35]. However, the molecular weight of the gM protein in our study is higher than its theoretical size of 45 kDa. To determine the cause of this unexpectedly high molecular weight of gM protein, the gM expression plasmid was sequenced, but no mutations were found. Furthermore, post-translational glycosylation was also ruled out with Endo H treatment, where the molecular weight of gM remained unchanged after treatment (data not shown). Further investigation on how post-translational modifications such as phosphorylation affected the size of gM protein will be warranted.

**Figure 2:**
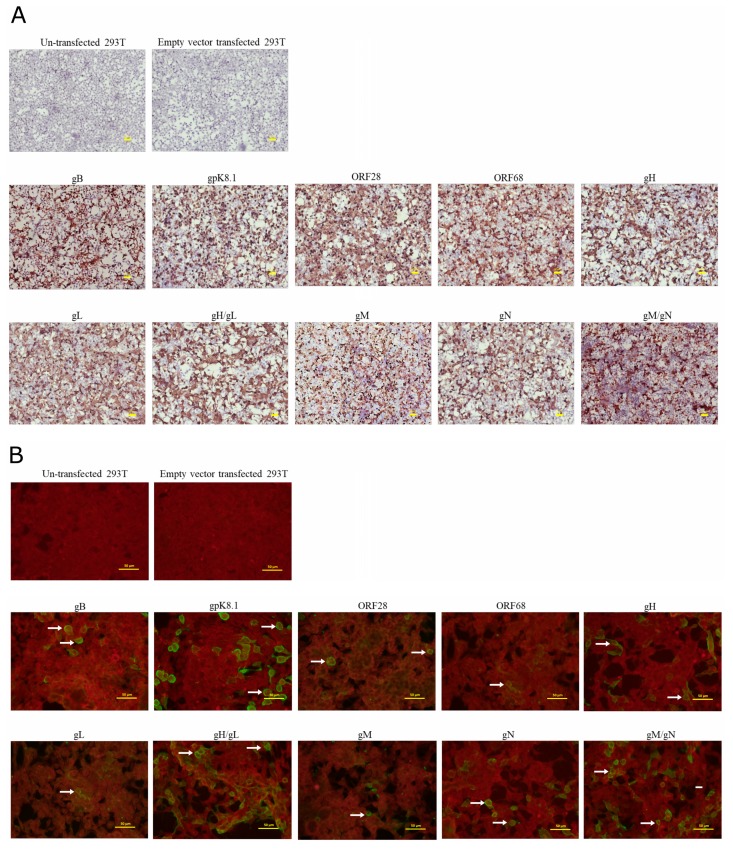
KSHV envelope glycoproteins expression in 293T cells by immunohistochemistry IHC and immunofluorescence assay (IFA). (**A**) KSHV glycoproteins expression was confirmed in 293T cells at 72 h post-transfection by IHC against the 3xFLAG tag. Positive cells are stained brown. (**B**) The cell surface expression of KSHV glycoproteins was confirmed by IFA on nonpermeabilized 293T cells at 72 h post-transfection with pooled plasma from all study participants. Positive cells are indicated by white arrows. Pictures were taken at 20X magnification.

To further confirm the expression and transfection efficiency for each glycoprotein and to demonstrate that the majority of the transfected cells indeed express the glycoproteins, immunohistochemistry (IHC) staining against the 3xFLAG tag was performed on the transfected cell cultures in situ at 72 h post-transfection. Since previous studies suggested that gH and gL form a noncovalent complex that is necessary for efficient transport of gL to the cell surface, cotransfection of gH/gL was also investigated [36]. Likewise, due to the previous reports that gN is required for functional processing of gM and together they form heterodimer, co-infection of gN/gM was included as well [37]. In agreement with the immunoblot results, the IHC demonstrated intense brown-color-stained cells, indicating high protein expression for all the KSHV glycoproteins in transfected cells (Figure 2A). Importantly, the majority of the cells in the transfected culture expressed their respective KSHV glycoprotein, demonstrating high transfection efficiency of each KSHV glycoprotein-encoding plasmid. Nontransfected and empty-vector-transfected 293T controls produced no evident chromagen deposition.

Additionally, to demonstrate that KSHV glycoproteins are indeed expressed on the transfected cell surface, IFA was performed on nonpermeabilized 293T cells at 72 h post-transfection using a pooled plasma from all the study participants (*n* = 25) as the primary detection antibody. The cell surface expression of all the KSHV glycoproteins was confirmed by the presence of green-colored cells (Figure 2B). Among the glycoproteins, the signal for ORF28, ORF68, gL alone and gM alone were relatively weak, which could be due to low levels of antibodies against these proteins in the pooled plasma. However, the combinations of gH/gL or gM/gN have relatively stronger green signal in comparison to respective single-plasmid-transfected cells, indicating that interaction between gH/gL or gM/gN potentiates recognition of these complexes by KSHV-infected plasma [36,37]. No signal was detected in the nontransfected and empty-vector-transfected 293T cells.

### 3.3. Antigenic Determinant of KSHV-Specific nAbs in the Plasma of KSHV-Infected Individuals

To determine which KSHV glycoprotein(s) are the target for KSHV-specific nAbs in the plasma of the non-KS asymptomatic individuals and KS patients, 293T cells expressing various KSHV glycoproteins were incubated with their plasma (*n* = 25) to adsorb nAbs against specific glycoproteins. The residual neutralizing capability of the depleted plasma was then quantified in a flow-cytometry-based neutralization assay. If nAbs against a specific KSHV glycoprotein were prevalent in a plasma, depletion by the 293T cells expressing that particular glycoprotein would result in a reduction in the ability of the depleted plasma to neutralize. These neutralization titers were then normalized with the percent neutralization of the identical plasma absorbed with empty vector-transfected cells and reflected as a relative percent nAbs absorbed.

**Figure 3:**
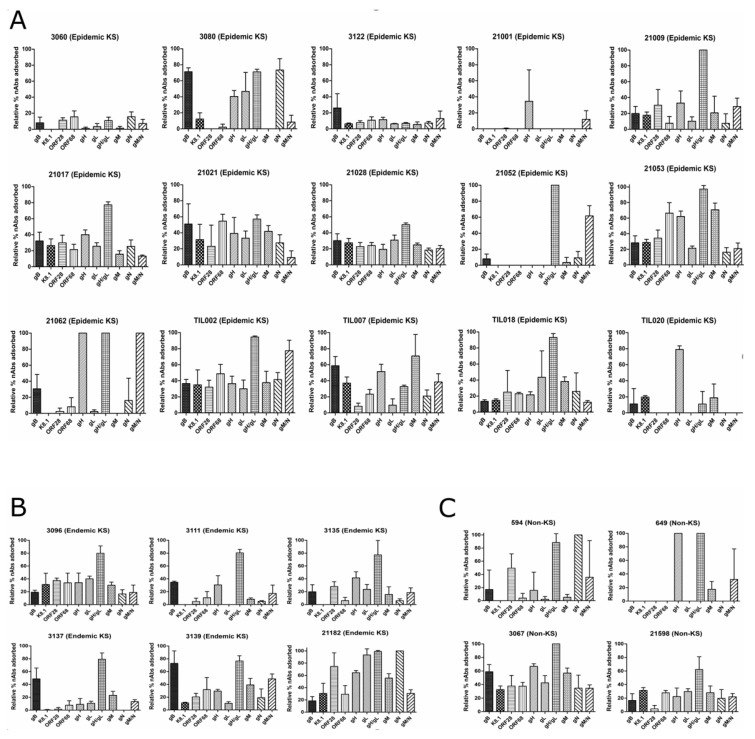
Antigenic determinant of KSHV-specific neutralizing antibodies (nAbs) in the plasma of KSHV-infected individuals. Heat-inactivated plasma of the study participants was absorbed with KSHV glycoproteins-expressing 293T cells, and neutralization assays were carried out as described in material and methods. The relative percentage of nAbs absorbed is shown for (**A**) epidemic KS (EpKS) cases, (**B**) endemic KS (EnKS) cases and (**C**) non-KS (asymptomatic KSHV-infected ) cases.

**Figure 4:**
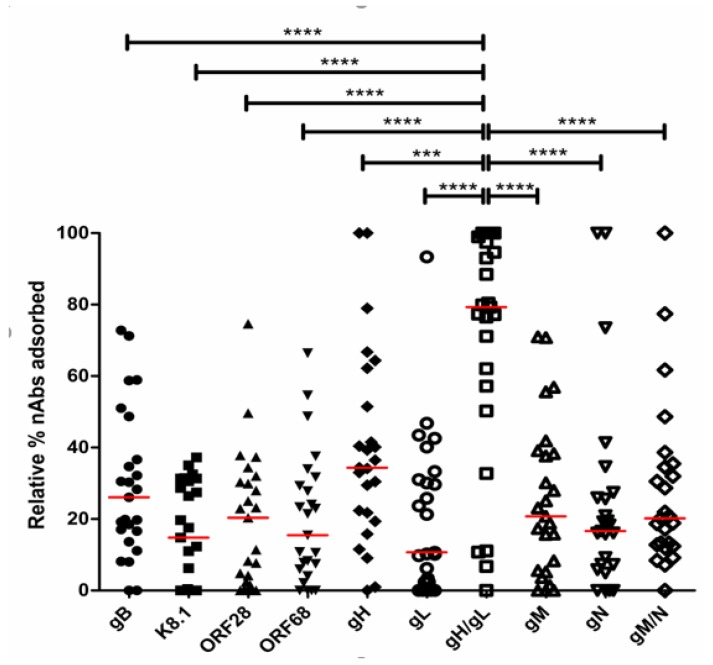
Comparison of nAbs responses among various KSHV envelope glycoproteins. The gH/gL complex-specific nAbs response was compared with other KSHV glycoproteins among all the participants using the Mann–Whitney test (*** *p* < 0.001, **** *p* < 0.0001).

Among the 15 EpKS cases, 80% of the patients’ plasma contained nAbs that could recognize eight out of 10 variations of the KSHV glycoproteins examined (Figure 3A). Only patients 21001, 21052 and TIL020 had nAbs that recognized less than 50% of the KSHV glycoproteins. Despite this breadth of KSHV glycoprotein recognition, the magnitude of neutralization attributable to each glycoprotein varied widely among individuals. For instance, there was a higher level of ORF28-specific nAbs in patient 21017 as compared to patient 3122. Similar patterns of nAbs breadth and magnitude were detected in the six EnKS and four non-KS cases (Figure 3B,C, respectively). Interestingly, among the entire cohort, only the gH/gL complex was consistently recognized by the majority of patients nAbs at high levels. In comparison to other KSHV glycoproteins, the near 80% response to gH/gL was significantly higher than that against all other glycoproteins or complexes (*p*-values ranged *p* < 0.001 to *p* < 0.0001) (Figure 4). Additionally, there is no correlation between the gH/gL-specific nAbs and the total anti-KSHV and total KSHV-specific nAbs responses, despite the fact that 80% of our study participants had demonstrated high level of nAbs against the gH/gL complex (Supplementary Figure S3A,B). Furthermore, no correlation was found between the nAbs responses against gH/gL complex and age, sex or symptomatic KS duration in the cohort (Supplementary Figure S3C–E).

### 3.4. Similar nAbs Responses towards KSHV Glycoproteins Regardless of KS Status

**Figure 5:**
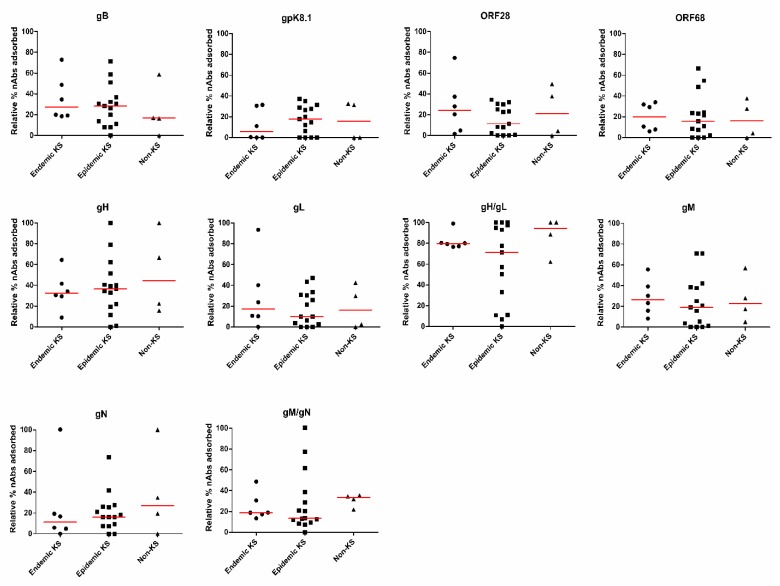
Comparison of nAbs responses to each KSHV glycoprotein in the EnKS, EpKS and non-KS groups. The nAbs responses against each KSHV glycoproteins were compared between the EnKS, EpKS and non-KS individuals using the Mann–Whitney test.

Our lab has previously shown that the magnitude of total KSHV nAbs responses are indistinguishable between EpKS and EnKS but are significantly higher in KS patients than non-KS individuals [34]. To determine if these observations extend to the KSHV glycoprotein-specific nAbs responses, the relative percent of nAbs depleted by each KSHV glycoprotein was compared between the EpKS, EnKS and non-KS groups. Unlike the total KSHV nAbs responses, the KSHV glycoprotein-specific nAbs responses were not statistically different between KS and non-KS groups (Figure 5). Thus, although the development of KS increased the magnitude of total nAbs in symptomatic patients, it had no detectable effect on the breadth and glycoprotein specificity in that increased response. Additionally, co-infection with HIV-1 seems to have no significant effect on the nAbs responses.

## 4. Discussion

Neutralizing antibodies are a powerful defense against many viral infections in animals and humans [38]. The nAbs against infections caused by viruses, such as influenza and flavivirus, have been shown to prevent viral infection; thus, it suggests that a similar approach may also be feasible in preventing KSHV infection [39,40,41,42]. Although KSHV-specific nAbs did not prevent the development of KS, whether the presence of these nAbs prior to exposure to KSHV would prevent infection is still unclear [13].

Since KSHV envelope glycoproteins are incorporated into the viral surface and are critical for viral entry into permissive cells, they are the candidate antigens for eliciting nAbs through vaccination. In recent years, several studies have shown that immunizing mice or rabbits with Newcastle disease virus-like particles (VLP) expressing KSHV gpK8.1, gB and gH/gL, either alone or together, can elicit nAbs responses against KSHV [16,17]. However, whether these glycoprotein-targeted responses are consistent with those in KSHV-infected individuals with strong neutralizing responses has not yet been investigated. Addressing this question could provide insights for the design of prophylactic vaccines against KSHV.

Our study is the first to define the specificities of nAbs responses within KSHV-infected individuals’ plasma against KSHV envelope glycoproteins. In addition, ours is the first study to investigate whether differentials in magnitude or breadth of nAbs specificities exist between EnKS, EpKS and non-KS individuals. To ensure that our results were not affected by the different KSHV glycoproteins expression, an equal copy number of each glycoprotein-encoding plasmid was transfected into equal number of cells, which was then used for the adsorption of nAbs from sample plasma with optimized number of cells, time and incubation temperature (Supplementary Figure S1B,C). A good example to support our conclusion that the protein expression did not affect the adsorption result is gpK8.1, which were highly expressed in transfected cells based on the immunoblot, IHC and IFA data, but only a small amount of nAbs was adsorbed by it. Using our optimized assays, we found that the neutralizing component of plasma in a majority of the study participants targeted multiple KSHV envelope glycoproteins. Although these glycoproteins can elicit nAbs in infected individuals, the magnitude of the neutralization response varies widely among individuals with some glycoproteins eliciting much stronger response in one individual than another. This strongly indicates that nAbs responses elicited by any KSHV envelope glycoprotein are highly individualized.

Among all the KSHV glycoproteins, only the gH/gL complex elicited nAbs responses that are consistent and comparable among most individuals. Eighty percent of study participants showed high levels of nAbs against the gH/gL complex. Given this consistently strong nAbs reaction against gH/gL from different individuals, we speculate that gH and gL may undergo conformational changes when complexed, which then expose yet-to-be-determined epitopes that are highly immunogenic, resulting in its high recognition by over 80% of the infected individuals. Interestingly, several individuals, such as patients 21062 and TIL002, had nAbs that recognized both gH/gL and gM/gN, while other individuals also have similar nAbs among different glycoproteins. One possible explanation for this observation could be that some nAbs are targeting the carbohydrate moieties, which might be common among different KSHV envelope glycoproteins. For example, studies have shown that KSHV gB is associated with high levels of mannose which could be targeted by KSHV-specific nAbs, but only in some individuals [43]. Another possibility is the presence of nAbs that recognize certain yet-to-be-determined conformational epitopes that may only be exposed when the KSHV glycoproteins interact with one another. This is feasible if these glycoproteins are in close proximity with one another on the virion surface. Unfortunately, there is very little information on the KSHV glycoprotein homo- or hetero-oligomeric structures and certainly none for those on the KSHV virion; therefore, more studies will be needed to support our observations.

Although our previous finding indicated that the total KSHV nAbs responses are similar in EnKS and EpKS, and that these responses are higher compared to asymptomatic individuals, we found no evidence for such differences in terms of nAbs specificities against different KSHV glycoproteins [34]. Thus, it would appear that the diversity of KSHV glycoproteins-specific nAbs responses is independent of KS disease progression, whereas the magnitude of the total KSHV nAbs response increases with disease progression, and both are independent of HIV-1 co-infection. We also did not find any correlation between KSHV glycoprotein-specific nAbs responses and the presence of KSHV virions in the plasma at the time of sampling. This may indicate that there is a tissue reservoir that is providing the necessary antigens to elicit humoral responses or that these are anamnestic responses from previous episodes of higher plasma viral load.

In summary, our data suggest that the KSHV glycoproteins gH/gL complex is the most prominent target for elicitation of nAbs in KSHV-infected individuals regardless of KS status. Thus, the KSHV glycoproteins gH/gL complex should be considered as a potential antigen in future KSHV prophylactic vaccine design.

## Figures and Tables

**Table 1:** Characteristics of the study cohort.

	Patient ID	Sex	Age	KS Months	HIV-1 Months	HIV-1 Plasma Viral Load [copies/mL]	ARV Months	1/IC_50_	IFA	Plasma ORF26 PCR
Endemic KS (KSHV+, HIV-1−)	3096	M	27	3	NA	NA	NA	395.1	20480	−
	3111	M	24	7	NA	NA	NA	438.9	5120	−
	3135	M	25	12	NA	NA	NA	1672.4	5120	−
	3137	M	51	NI	NA	NA	NA	292.2	5120	−
	3139	M	27	14	NA	NA	NA	281.8	40960	+
	21182	M	57	108	NA	NA	NA	115.7	10240	NI
Epidemic KS (KSHV+, HIV-1+)	3060	F	44	8	12	BDL	9	1259.4	40960	+
	3080	F	21	3	5	BDL	1	199.9	1280	−
	3122	M	34	3	24	BDL	24	2779.9	40960	+
	21001	F	36	18	168	BDL	10	605.3	2560	+
	21009	M	36	60	120	BDL	9	232.2	1280	−
	21017	M	38	24	36	BDL	36	845.7	1280	+
	21021	M	41	60	NI	1.16 × 10^5^	NI	1606.7	1280	+
	21028	M	30	24	24	BDL	24	1167.5	5120	−
	21052	F	37	84	NI	BDL	48	1971.7	2560	−
	21053	M	44	9	5	BDL	4	890.4	2560	−
	21062	M	68	24	NI	BDL	NI	715.8	5120	+
	TIL002	M	68	3	48	2201	36	285.2	5120	NI
	TIL007	M	46	72	72	33	72	407.1	5120	NI
	TIL018	M	55	48	120	BDL	120	1600.5	5120	NI
	TIL020	M	51	2	7	BDL	7	454.4	10240	NI
Non-KS (KSHV+, HIV-1-)	3067	F	60	NA	NA	NA	NA	224.6	5120	NI
Non-KS (KSHV+, HIV-1+)	21598	F	45	NA	36	NI	NI	197.1	2560	NI
	594	F	33	NA	18	NI	12	175.4	2560	NI
	649	F	25	NA	6	NI	4	126.1	640	NI

‘+’ denotes KSHV virions present in plasma, and ‘−’ denotes KSHV virions not present in plasma. Abbreviations: KSHV, Kaposi’s sarcoma-associated herpesvirus; KS, Kaposi sarcoma; HIV-1, human immunodeficiency virus type 1; ARV, Antiretrovirals; IFA, immunofluorescence assay; NA, not applicable; NI, no information; BDL, below detection limit. IFA numbers represent total anti-KSHV antibody titer (reciprocal endpoint plasma dilution); 1/IC50 numbers represent the dilution of plasma that has a 50% neutralizing activity. Green denotes Endemic KS cases. Red denotes Epidemic KS cases. Blue denotes Non-KS controls without or with HIV-1 infection.

## Supplementary Materials

The following are available online at https://www.mdpi.com/1999-4915/12/3/256/s1, Figure S1: Titration of rKSHV.219, optimization conditions for absorption of plasma with KHSV glycoprotein expressing 293T cells. (A) rKSHV.219 titration in 293T cells. 293T cells were seeded at a density of 2.5 × 10^6^ in triplicates into a 96-well plate. The cells were then infected with either 10, 20, 30, 40, 60 or 80 µl of concentrated virus in a total volume of 200 µL for 72 h at 37 °C. Infected cells (GFP+ cells) were quantified using FACS by acquiring a total of 10000 events. (B) Optimization of binding temperature during absorption of nAbs with gH/gL complex expressing 293T cells. (C) Optimization of the number of gH/gL complex expressing 293T cells during absorption with nAbs., Figure S2: Total KSHV-specific nAbs of the study participants. Neutralization assays were conducted by incubating rKSHV.219 with heat-inactivated plasma at 1:50 dilutions. The level of neutralization was determined after 72 h using flow cytometry to quantify the number of GFP+ cells acquiring a total of 10000 events. Figure S3: Comparison of the magnitude of nAb responses to KSHV gH/gL complex with; total anti-KSHV antibodies and with total KSHV-specific nAb responses, age, sex and symptomatic KS duration. The correlation between the extent of nAb absorbed by gH/gL and (A) total anti-KSHV and (B) nAb responses, (C) age, (D) sex and (E) symptomatic KS duration was performed using nonparametric Spearman correlation analysis (ns *P* > 0.05).
