# Supplementary material for: The Kaposi’s Sarcoma-Associated Herpesvirus (KSHV) gH/gL Complex Is the Predominant Neutralizing Antigenic Determinant in KSHV-Infected Individuals
Source: Viruses. 2020 Feb 26;12(3):256. doi: 10.3390/v12030256 (PMC7150787; doi:10.3390/v12030256)
Supplement: Supplementary file 1 [file viruses-12-00256-s001.pdf]

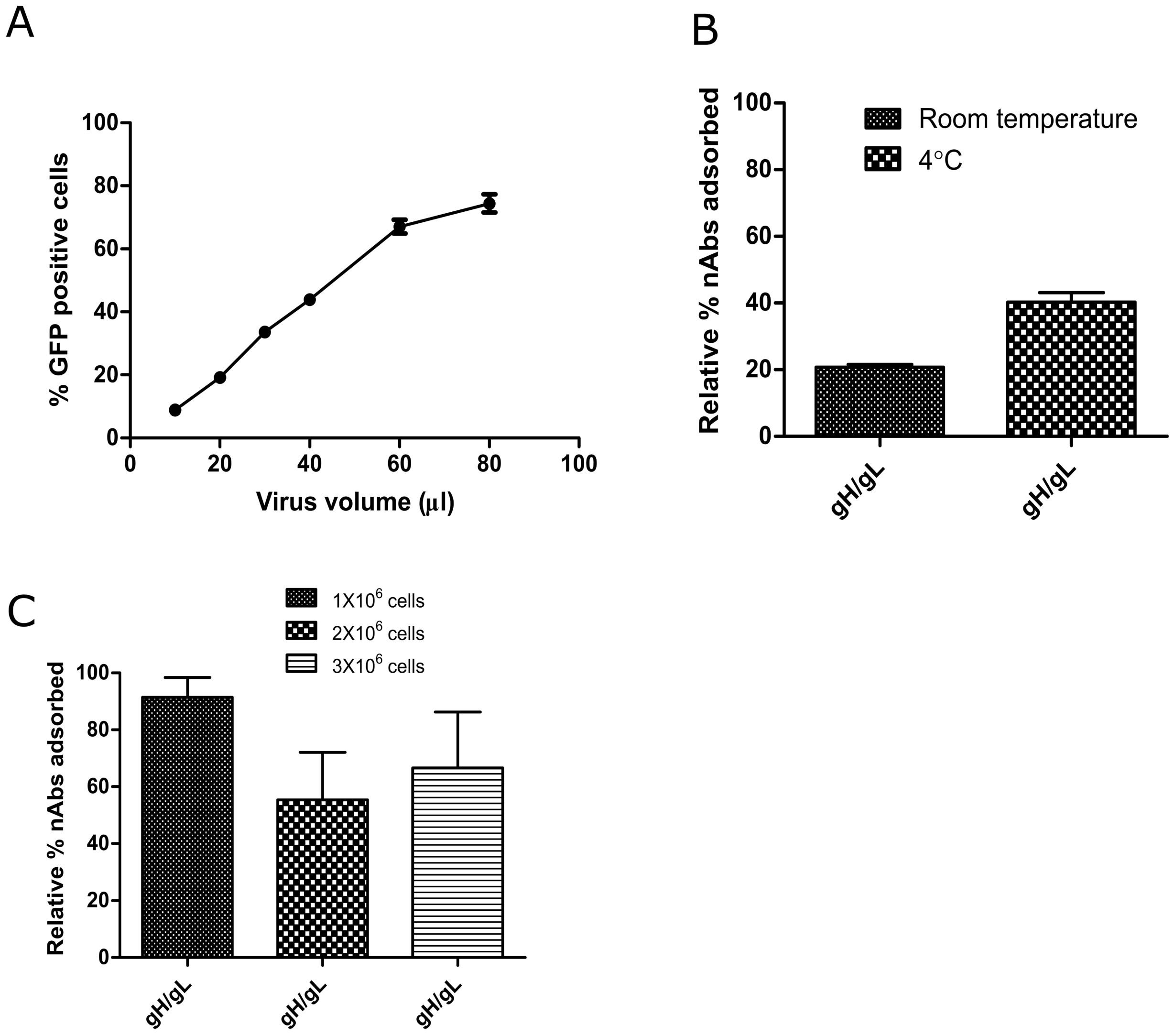

Figure S1. Titration of rKSHV.219, optimization conditions for absorption of plasma with KHSV glycoprotein expressing 293T cells. (A) rKSHV.219 titration in 293T cells. 293T cells were seeded at a density of 2.5 x 10^6 in triplicates into a 96-well plate. The cells were then infected with either 10, 20, 30, 40, 60 or 80 µl of concentrated virus in a total volume of 200 µl for 72 hours at 37°C. Infected cells (GFP+ cells) were quantified using FACS by acquiring a total of 10000 events. (B) Optimization of binding temperature during absorption of nAbs with gH/gL complex expressing 293T cells. (C) Optimization of the number of gH/gL complex expressing 293T cells during absorption with nAbs.

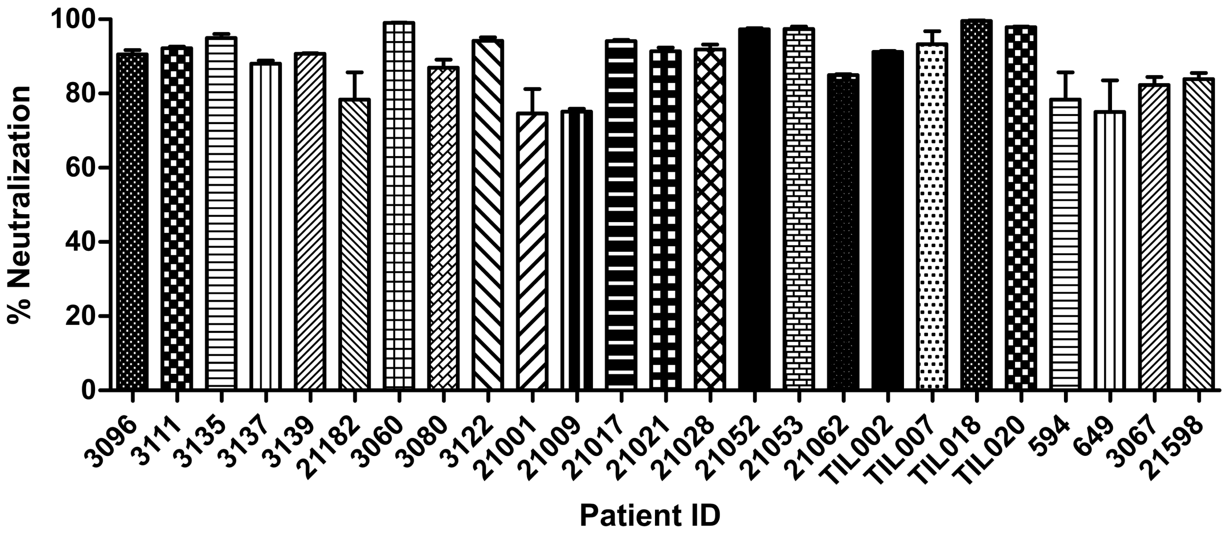

Figure S2. Total KSHV-specific nAbs of the study participants. Neutralization assays were conducted by incubating rKSHV.219 with heat-inactivated plasma at 1:50 dilutions. The level of neutralization was determined after 72 hours using flow cytometry to quantify the number of GFP+ cells acquiring a total of 10000 events.

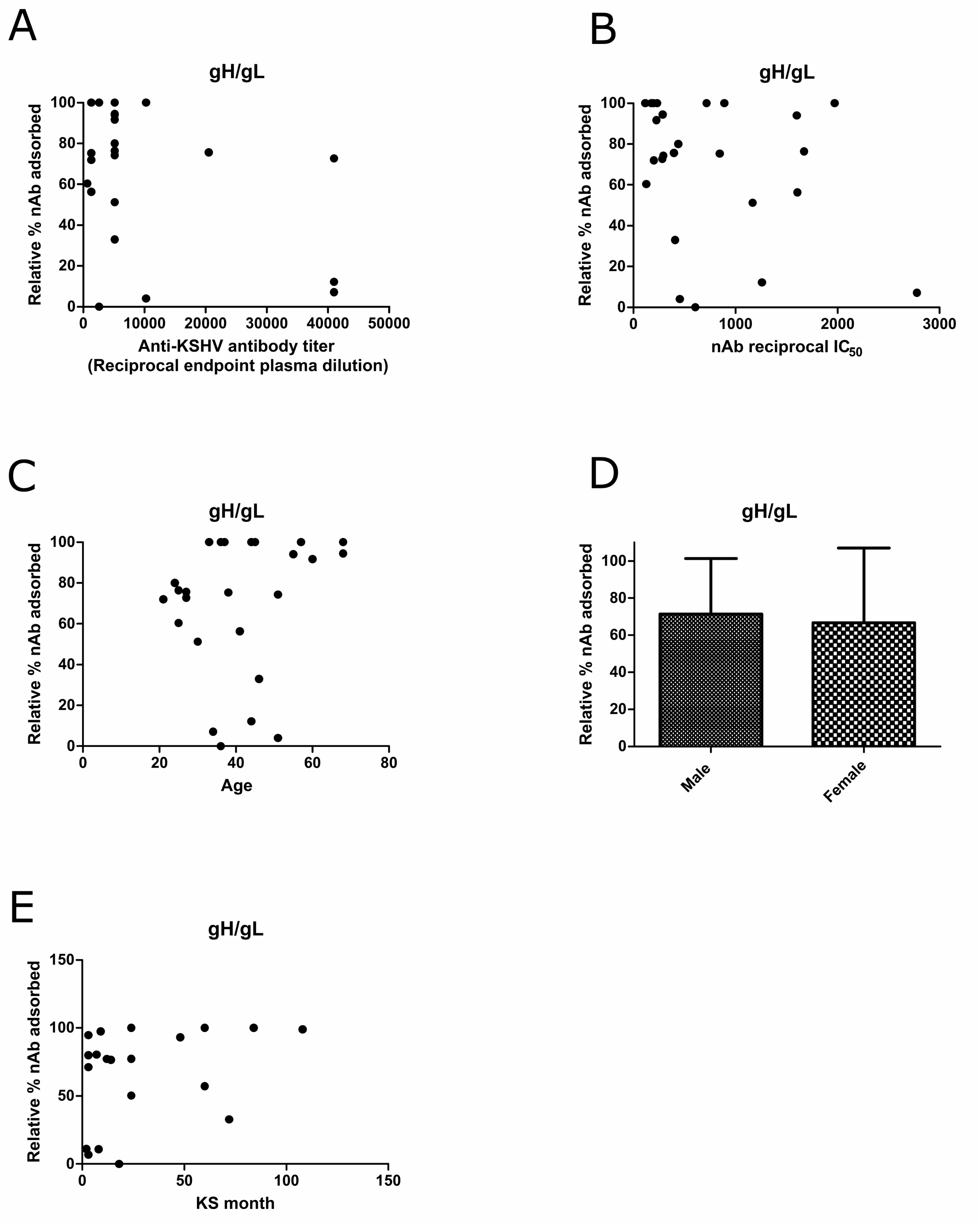

Figure S3. Comparison of the magnitude of nAb responses to KSHV gH/gL complex with; total anti-KSHV antibodies and with total KSHV-specific nAb responses, age, sex and symptomatic KS duration. The correlation between the extent of nAb absorbed by gH/gL and

(A) total anti-KSHV and (B) nAb responses, (C) age, (D) sex and (E) symptomatic KS duration was performed using nonparametric Spearman correlation analysis (ns *P*>0.05).
